# The Global Virome in One Network (VIRION): an Atlas of Vertebrate-Virus Associations

**DOI:** 10.1128/mbio.02985-21

**Published:** 2022-03-01

**Authors:** Colin J. Carlson, Rory J. Gibb, Gregory F. Albery, Liam Brierley, Ryan P. Connor, Tad A. Dallas, Evan A. Eskew, Anna C. Fagre, Maxwell J. Farrell, Hannah K. Frank, Renata L. Muylaert, Timothée Poisot, Angela L. Rasmussen, Sadie J. Ryan, Stephanie N. Seifert

**Affiliations:** a Department of Microbiology and Immunology, Georgetown University Medical Center, Georgetown University, Washington, DC, USA; b Center for Global Health Science and Security, Georgetown University Medical Center, Georgetown University, Washington, DC, USA; c Department of Biology, Georgetown University, Washington, DC, USA; d Centre for Mathematical Modelling of Infectious Diseases, London School of Hygiene and Tropical Medicine, London, United Kingdom; e Centre on Climate Change and Planetary Health, London School of Hygiene and Tropical Medicine, London, United Kingdom; f Department of Health Data Science, University of Liverpool, Liverpool, United Kingdom; g National Center for Biotechnology Information, U.S. National Library of Medicine, National Institutes of Health, Bethesda, Maryland, USA; h Department of Biological Sciences, Louisiana State University, Baton Rouge, Louisiana, USA; i Department of Biological Sciences, University of South Carolina, Columbia, South Carolina, USA; j Department of Biology, Pacific Lutheran University, Tacoma, Washington, USA; k Department of Microbiology, Immunology, and Pathology, Colorado State University, Fort Collins, Colorado, USA; l Bat Health Foundation, Fort Collins, Colorado, USA; m Department of Ecology & Evolutionary Biology, University of Toronto, Toronto, Ontario, Canada; n Department of Ecology and Evolutionary Biology, Tulane University, New Orleans, Louisiana, USA; o Molecular Epidemiology and Public Health Laboratory, Hopkirk Research Institute, Massey University, Palmerston North, New Zealand; p Département de Sciences Biologiques, Université de Montréal, Montréal, Quebec, Canada; q Québec Centre for Biodiversity Science, Montréal, Quebec, Canada; r Vaccine and Infectious Disease Organization, University of Saskatchewan, Saskatoon, Saskatchewan, Canada; s Department of Geography, University of Florida, Gainesville, Florida, USA; t Emerging Pathogens Institute, University of Florida, Gainesville, Florida, USA; u School of Life Sciences, University of KwaZulu-Natal, Durban, South Africa; v Paul G. Allen School of Global Health, Washington State University, Pullman, Washington, USA; Brigham Young University; University of Pennsylvania

**Keywords:** data synthesis, ecological networks, global virome, host-virus interactions

## Abstract

Data that catalogue viral diversity on Earth have been fragmented across sources, disciplines, formats, and various degrees of open sharing, posing challenges for research on macroecology, evolution, and public health. Here, we solve this problem by establishing a dynamically maintained database of vertebrate-virus associations, called The Global Virome in One Network (VIRION). The VIRION database has been assembled through both reconciliation of static data sets and integration of dynamically updated databases. These data sources are all harmonized against one taxonomic backbone, including metadata on host and virus taxonomic validity and higher classification; additional metadata on sampling methodology and evidence strength are also available in a harmonized format. In total, the VIRION database is the largest open-source, open-access database of its kind, with roughly half a million unique records that include 9,521 resolved virus “species” (of which 1,661 are ICTV ratified), 3,692 resolved vertebrate host species, and 23,147 unique interactions between taxonomically valid organisms. Together, these data cover roughly a quarter of mammal diversity, a 10th of bird diversity, and ∼6% of the estimated total diversity of vertebrates, and a much larger proportion of their virome than any previous database. We show how these data can be used to test hypotheses about microbiology, ecology, and evolution and make suggestions for best practices that address the unique mix of evidence that coexists in these data.

## INTRODUCTION

The global virome—the aggregate of all viruses across the entire biosphere—is one of the most underdocumented components of global biodiversity. There are at least 40,000 species of viruses estimated to infect mammals alone, of which thousands can probably infect humans (1); thousands of others, if not millions, are distributed across the tree of life, of which a select few can cross between branches with a deep evolutionary split. For example, both influenza viruses and coronaviruses circulate among birds, terrestrial mammals, and cetaceans (2); similarly, West Nile virus infects not only humans and other mammals but also birds (3) and even some reptiles and amphibians (4). In this regard, the global virome forms a broad, tangled network of species interactions—a network that is substantially underdocumented.

To date, most data sources that describe the animal-virus network have been limited to a small proportion of known mammal viruses, covering at most 1 to 2% of the total viral diversity in this class; data on the other vertebrate classes are even more limited. These data are a critical piece of zoonotic risk assessment, as viruses capable of broad host jumps are those most predisposed to future emergence. Moreover, recent evidence shows that data on the network structure of the global virome can be used to improve predictions of viruses with zoonotic potential based on genome composition (5). Despite the obvious value of host-virus network data, currently available data sets are notably limited by the challenges of data integration and reconciliation; manual recompilation and reconciliation are too inefficient to consistently keep these data sets up to date, and many sources are effectively a decade behind current knowledge (6). Therefore, open, reproducible data that are as complete and detailed as possible are desperately needed.

**FIG 1:**
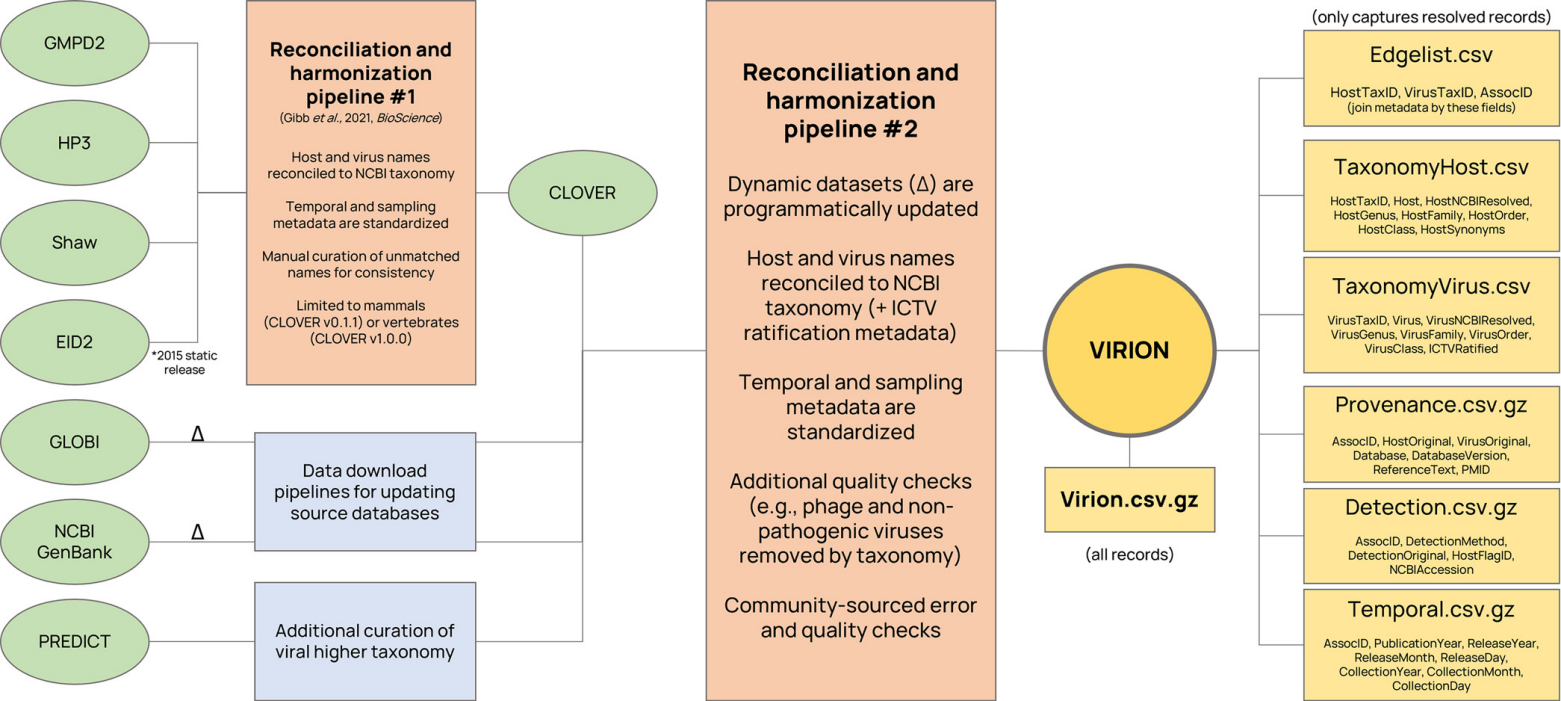
The VIRION pipeline. Data are integrated from a total of seven sources into one master file and a set of six disaggregated files that constitute the VIRION database. Data sources marked with a delta can be dynamically updated as new data are submitted to the source databases.

Here, we describe a new open database called The Global Virome in One Network (VIRION), which integrates different existing data sets to produce the most comprehensive picture ever developed of the global vertebrate virome. A total of seven data sources on host-virus associations (evidence that a virus is capable of infecting, or is known to infect, a given host) are integrated in a pipeline that standardizes taxonomy and harmonizes sampling metadata (Fig. 1). The current version of the data product, and disaggregated data sets for more informal inquiry, are all openly available with this publication.

## RESULTS AND DISCUSSION

### The assembled VIRION database.

 

**(i) The current scope of the data.** VIRION is the most comprehensive open-access database characterizing the vertebrate virome. Version 0.2.1 of the database (a stable release that accompanies this study) contains a total of 484,464 records. The majority of distinct records (*n* = 391,487; ∼81%) come from GenBank, particularly due to human samples with distinct dates of a few very well sampled viruses (e.g., influenza A virus, HIV-1, SARS-CoV-2, and Norwalk virus). The remaining 20% or so of the data are distributed unevenly among EID2 (*n* = 57,265; 12%), GLOBI (19,661; 4%), Shaw (8,643; 2%), PREDICT (2,850; <1%), HP3 (2,802; <1%), and GMPD2 (1,765; <1%). After removing metadata, distinct values of resolved host-virus associations are more evenly distributed among GenBank (15,467; 36%), GLOBI (13,924; 33%), Shaw (6,418; 15%), HP3 (2,759; 6%), EID2 (2,025; 5%), PREDICT (952; 2%), and GMPD2 (939; 2%).

**FIG 2:**
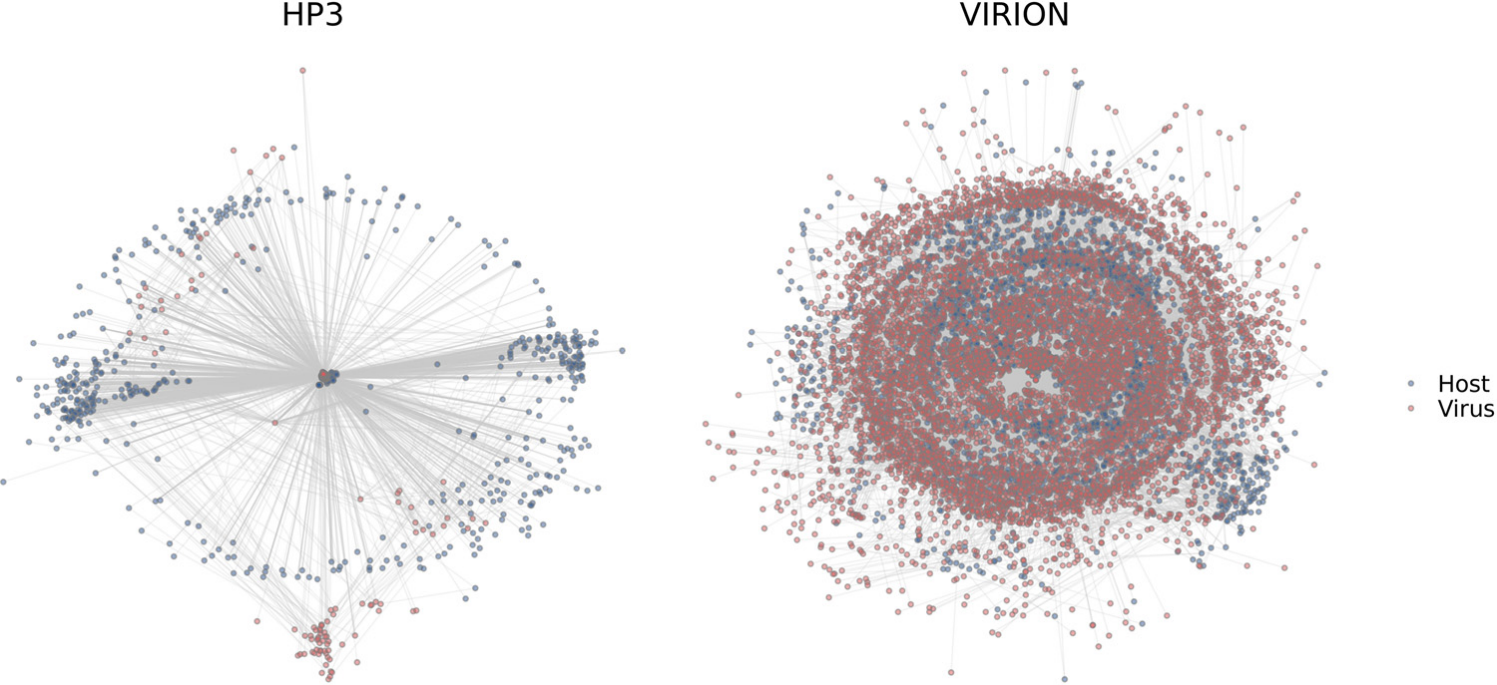
Comparative scope of data. Networks show all unique NCBI-recognized host-virus species pairs (viruses are red, hosts are blue) in the Host-Pathogen Phylogeny Project database (HP3, published in 2017) and VIRION. The information stored in VIRION is more extensive (including all vertebrates, not just mammals) but also far more information dense, describing a network with many more nodes and many more connections.

**FIG 3:**
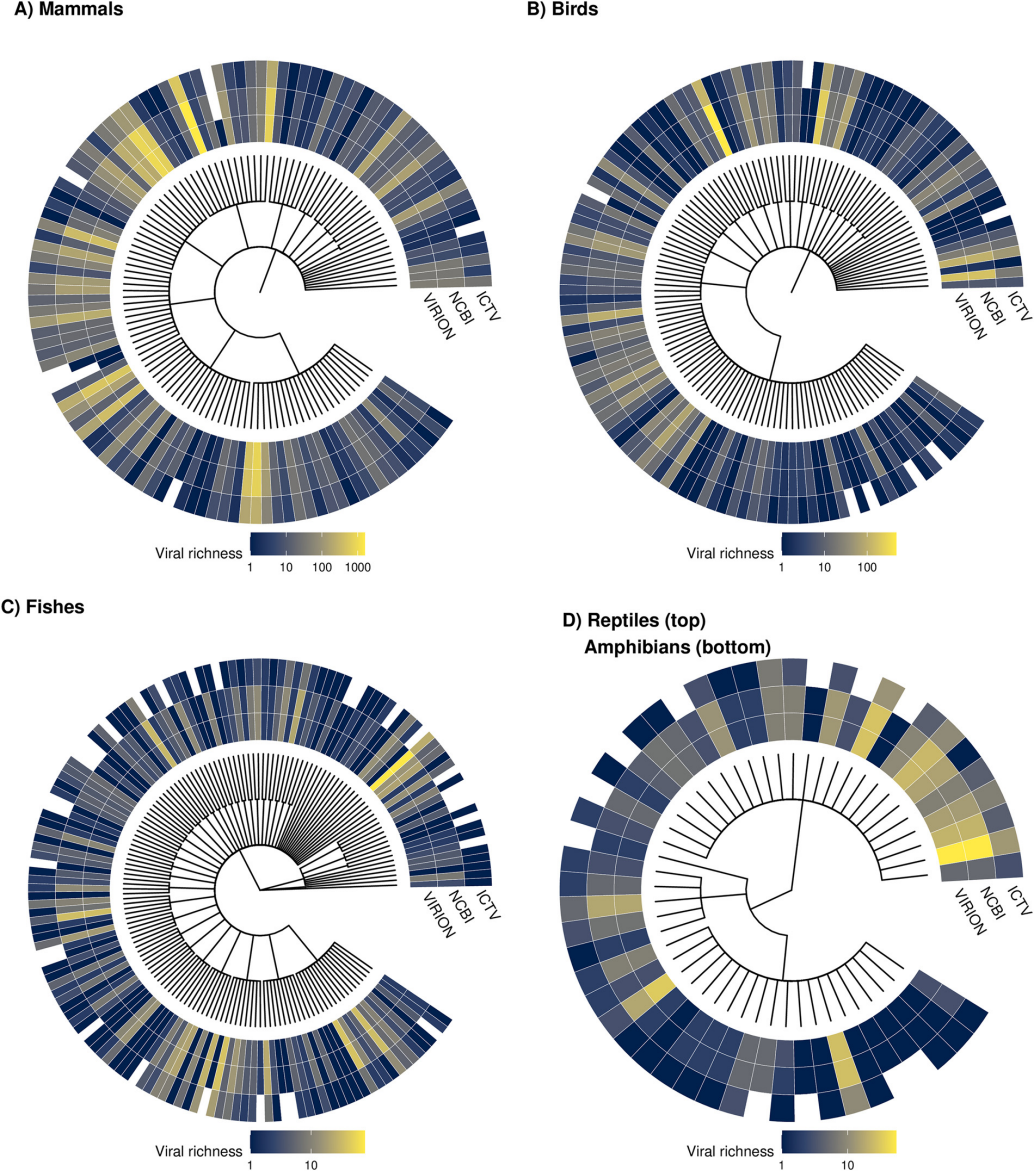
Taxonomic coverage across hosts. Each tree tip represents one host family, with the total number of viruses recorded in VIRION, the number that are NCBI resolved, and the number that are ICTV ratified. Note that the color scale varies across panels.

**FIG 4:**
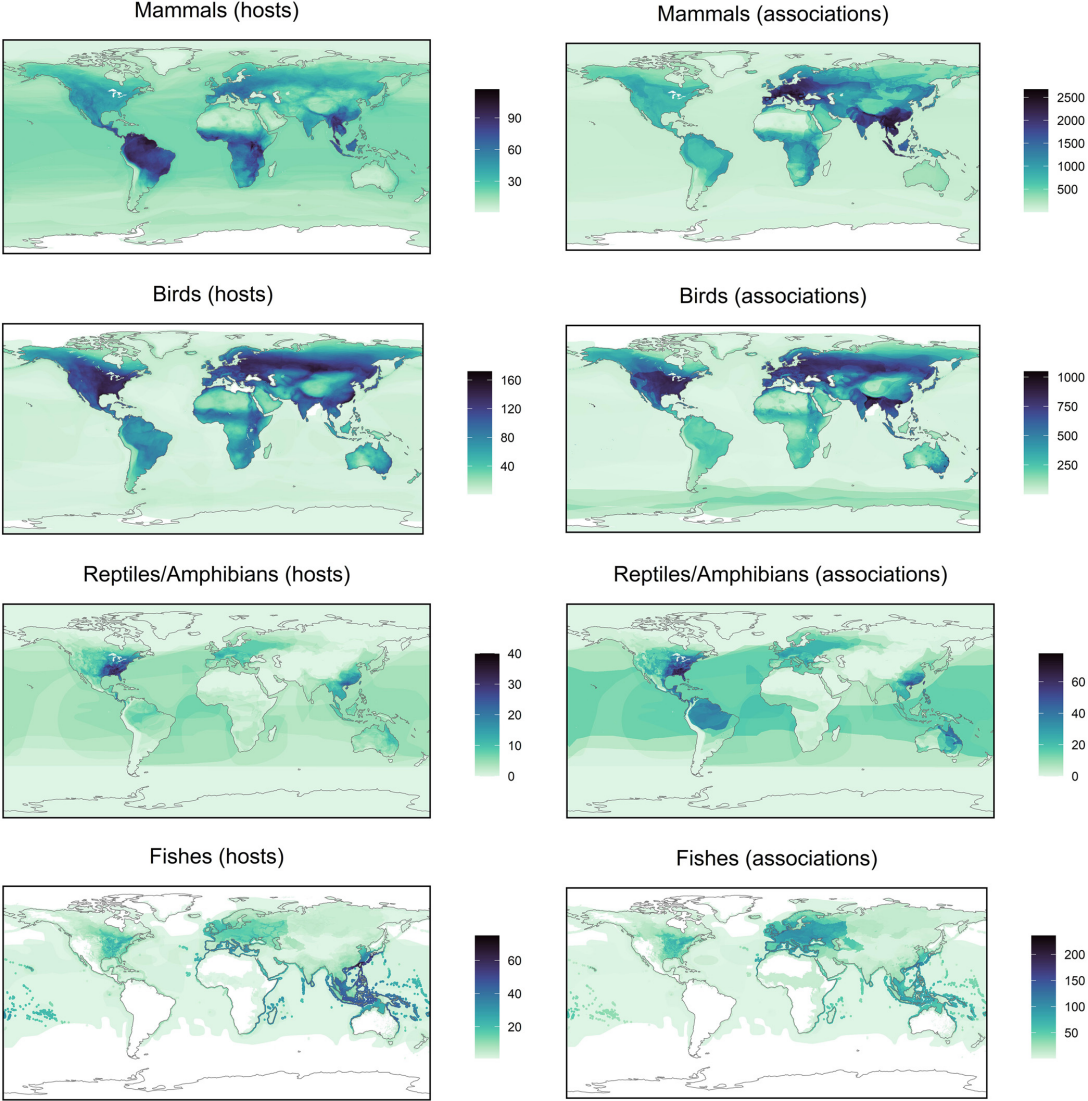
The geographic distribution of hosts and host-virus associations based on IUCN geographic range maps. Species are matched to the IUCN database using verbatim Latin names, without any manual correction. This is largely congruent for mammals (91.1%) and birds (93%) but less so for reptiles/amphibians (79%) and fish (47.6%), in part because some species may not yet be mapped. Particularly when working with the latter groups, users will likely need to manually cross-reference species names from the VIRION database to other sources.

The VIRION database is far more extensive than many of these source data sets on their own and is information dense with respect to associations and not just species (Fig. 2). The association data include 9,521 resolved virus “species” (of which 1,661 are ICTV ratified), 3,692 resolved vertebrate host species, and 23,147 unique interactions between taxonomically valid organisms. Together, these data cover ∼25% (1,635 species) of mammal diversity (∼6,500 species), ∼11% (1,072 species) of birds (∼10,000 species), and ∼6% of the estimated total diversity of vertebrates (∼60,000 species). Each of these groups has its own unique coverage biases and gaps in terms of both host taxonomy (Fig. 3) and geographic sampling (Fig. 4), which are nontrivial enough to shape the findings of research that uses the database.

### (ii) How the database updates.

New viruses are constantly discovered, and existing viruses are often found in unexpected hosts. As records of new host-virus associations are published, updates to VIRION will reflect this expanding knowledge base. At present, the database is automatically updated using GitHub Actions; on a nightly basis, the dynamic sources (GenBank and GLOBI) are redownloaded, undergo full taxonomic reconciliation, and are reincorporated into the full database. The pipeline can also be manually executed in full to update the taxonomy in the static sources (CLOVER and PREDICT), producing a new stable build.

### How to use VIRION.

Both automatic and stable builds can be downloaded from a web portal with both automated and manually compiled versions (viralemergence.github.io/virion), or directly from the open-source, open-access, version-labeled GitHub repository (github.com/viralemergence/virion). While the database can be used fairly out-of-the-box thanks to the taxonomic reconciliation pipeline, we encourage users to think carefully about the data they are manipulating and how it relates to their analysis. Here, we recommend some simple steps toward that end and show some example uses (based on release version 0.2.1).

### Some best practices.

We recommend that users should consider the following steps as part of their workflow:

1. **Be aware of the challenges of big data.** Make sure that column types are read in correctly if columns are particularly sparse, be aware of the challenges of reading and writing large files (packages like ‘data.table’ and ‘vroom’ in R or ‘DataFrames.jl’, ‘JuliaDB.jl’, and ‘Arrow.jl’ in Julia help with this), and note the small handful of optimizations we have made to make VIRION easier to work with (in particular, NCBI accession values are stored as comma-space separated lists—e.g., “KP272011.1, KP272012.1”—for records that are otherwise identical in all other metadata).
2. **Check for quality issues programmatically.** For example, “HostFlagID” denotes the presence of possible uncertainty in host identification, which users may want to check before proceeding any further. This uncertainty may or may not be acceptable depending on data provenance and user intentions.
3. **Check for quality issues manually when possible.** For the most important applications, narrowly defining the data set of interest can help users make the task of manual quality control a manageable one. For example, if a researcher is developing a predictive model of the host-virus network for the purpose of identifying viruses with zoonotic potential, the researcher may want to manually check each virus that is recorded as having human hosts (SEE EXAMPLE 2), which will be the most important subset of the data for model performance.
4. **Consider the underlying biology.** Not every data point necessarily represents a real host-virus interaction, even when a virus has been found inside a host. While our data set currently excludes all bacteriophages, other associations may still be problematic. The family *Flaviviridae*, for example, includes a mix of vertebrate viruses (e.g., hepatitis C virus), mosquito- and tick-borne viruses (dengue virus and tick-borne encephalitis virus), and insect-specific viruses (Palm Creek virus). If an animal has consumed infected insects, these viruses might be discovered in vertebrate samples, and—before viruses are characterized (e.g., phylogenetic placement, dinucleotide composition analysis, cell culture)—it may not be immediately obvious which viruses are insect specific. Similar problems exist for rhabdoviruses, which are increasingly known to include insect-specific viruses, and picobirnaviruses, which are increasingly understood to infect prokaryotes. In the future, it may also be easier to distinguish these viruses based on sampling methodology (e.g., insect viruses may be found in vertebrate fecal samples but absent from their tissues), if these metadata can be systematically recovered.
5. **Consider data origins and sampling.** The data sets in CLOVER contain rich information on which associations are known from serology, sequencing/PCR, or viral isolation. Other data sources contain less-rich information: GLOBI does not retain this sampling metadata, and all GenBank records are assigned to sequencing as the “minimum evidence standard” even though some may be viral isolation. In combination with other nuances about data provenance, this can also indicate differences in the underlying biological reality: for example, HP3, Shaw, and GMPD2 are manually curated to include data only from wild, “natural” infections and explicitly exclude experimental infections; EID2, which automatically pulls in new records from sources like GenBank, contains some experimental infections (e.g., baboons and macaques in the United States). Similarly, users should note that the Virus-Host DB includes some host-virus associations inferred from cell line experiments, which cannot necessarily be easily identified within the database itself and which are reproduced downstream in GLOBI without the commented metadata that note this; as a result, users may find some surprising cases like anaconda paramyxovirus being reported as zoonotic. A researcher interested in host-virus biological compatibility may be fine using any of the data sources in VIRION, while one trying to document patterns in the wild may wish to subset based on detection and data provenance accordingly.
6. **Consider taxonomic resolution.** Most users will want to use only records where both the virus and host names are NCBI resolved; though these are relatively standardized, many still include sampling metadata or unclassified lineage information for viruses. Simple rubrics can flag these cases: for example, in the list of NCBI-accepted betacoronavirus names, searching for virus names that include a “/” will flag many lineage-specific records (“bat coronavirus 2265/philippines/2010,” “coronavirus n.noc/vm199/2007/nld”) and separate them from some cleaner names (“alpaca coronavirus”) but will not necessarily catch everything (“bat coronavirus ank045f”). Another option is to limit analysis to viruses that are ICTV ratified (“ICTVRatified” = TRUE), but this is particularly conservative and will leave a much larger number of valid virus names out (Fig. 3). Users should consider a readthrough of all of the species names in the data set they are working with, just to familiarize themselves with any remaining oddities. (When errors or oddities are detected in the NCBI taxonomy, they can be submitted for investigation and a possible patch on the GitHub repository; these issues should be tagged using the label “taxonomy.”)
7. **Consider paraphyletic relationships.** The NCBI taxonomic backbone uses no-rank “in part” names to denote paraphyletic groups; these ranks may not be found consistently at higher taxonomic levels that belong to the paraphyletic group. In a situation where a user would query all viruses for a paraphyletic group like Reptilia, this can lead to missing hosts. An alternative more robust practice would be to query the component taxa directly, i.e., Lepidosauria (rank: class; TaxID: 8504) and Testudines (order; 8459) and Crocodylia (order; 1294634). These issues are likely to be more prevalent at high taxonomic ranks in the host taxonomy.
8. **Consider reanalysis when necessary.** Given the challenges of viral taxonomy and the unique problems of synthetic data, some researchers may even want to use VIRION as a starting point for a more in-depth reanalysis. For example, a researcher interested in a particular viral family might use the linked metadata to download all available GenBank sequences, build a phylogenetic tree, and group sequences into operational taxonomic units (OTUs) based on their similarity. Specific data sets may particularly benefit from this thoroughness: for example, while the PREDICT project used taxonomic cutoffs for sequence dissimilarity based on ICTV rules, the sequences generated by the program are mostly short fragments; taxonomic definitions based on these short sequences and others using whole genomes, or targeting different regions, all coexist within VIRION.

Finally, we encourage users who discover unusual interactions to investigate their provenance and potentially flag controversial data in source data sets; this can be particularly important for the most valuable records in VIRION, such as those that document zoonotic infections. Erroneous records of human infection are likely to significantly impact models and may even lead to public health confusion, and as such, data should be carefully handled and reproduced in analytical settings.

### Example 1: where does chikungunya come from?

In the early days of the Verena Consortium (viralemergence.org), a debate started in the margins of a heavily edited manuscript: is it appropriate to include chikungunya in a list of zoonotic diseases? Chikungunya virus is a mosquito-borne alphavirus of particular concern to public health in the tropics; unlike many comparable alphaviruses (e.g., Mayaro virus) and flaviviruses (e.g., Zika virus), chikungunya virus is rarely thought of as a pathogen with a sylvatic cycle in nonhuman primates. But like most *Aedes-*borne viruses, it can certainly infect some animals, having been isolated from nonhuman primates (7), bats (7, 8), and birds (9) in the wild. Further, experimental challenge studies indicate susceptibility of additional bat species, amphibians, and reptiles to chikungunya virus (10–12). To investigate its naturally occurring host range, a user might begin by loading the data, subsetting to records about chikungunya virus, and examining the raw data:
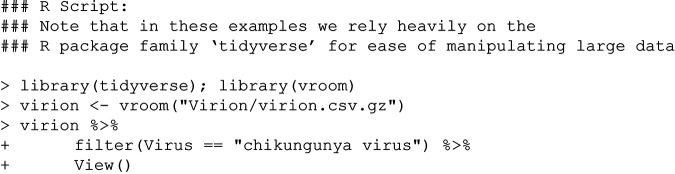


There are nearly 2,000 records for chikungunya virus in the VIRION database that are called up by this function. If a user was interested in investigating the host range contained in those records, the user might then call:
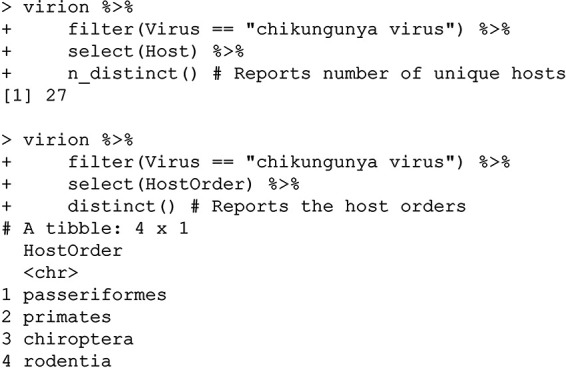


There are (at the time of writing) 27 unique hosts, including primates, bats, rodents, and—surprisingly—perching birds (passerines). Interested in following up on that finding, a user could simply call:
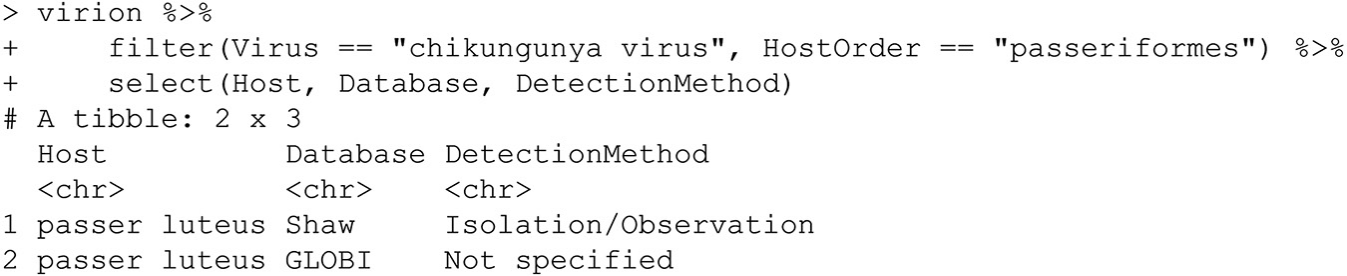


In most cases, viral isolation is taken as the most stringent possible evidence of host competence based on detection alone (13), implying that the Sudan golden sparrow (Passer luteus) or species like it could be part of the virus’s sylvatic cycle in Africa. (This example also highlights another key point about the VIRION architecture: because some data sources index others—in this case, because the Shaw database is indexed in GLOBI—there will be some redundancy between records. While this is to be expected, it can also lead to metadata loss; for example, here, the GLOBI records do not distinguish viral isolation from PCR or serology.) Despite this interesting bird record, mammals seem a more likely sylvatic host on balance (though the distribution of research effort has also probably been uneven):
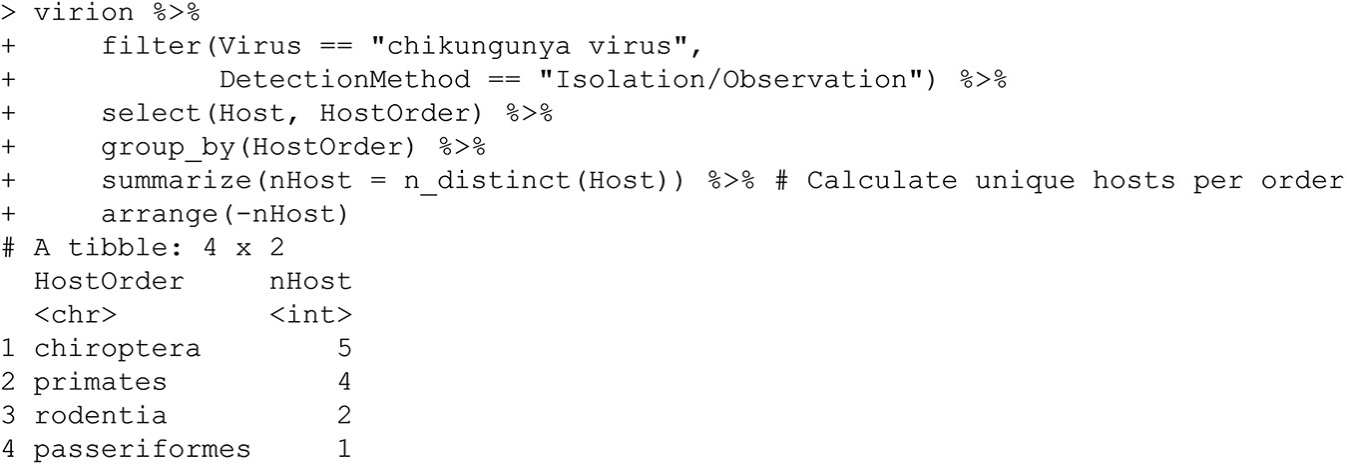


While mosquito-borne alphaviruses like Mayaro virus are usually thought of as having primate reservoirs, these results suggest that closer attention might be required with regard to the role that bats play in the circulation of chikungunya—an idea with broader support for other arboviruses (14).

### Example 2: do fish host any zoonotic viruses?

Most zoonotic viruses are hosted by mammals, while a few have bird reservoirs; only a small number can infect other vertebrate classes. Are there any truly zoonotic viruses that can also infect fish? To answer this question, we can first generate a list of viruses that are recorded as infecting humans:
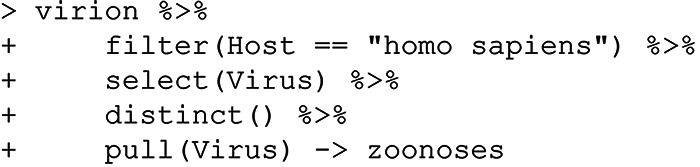


Next, we pull all records of those viruses in fish:
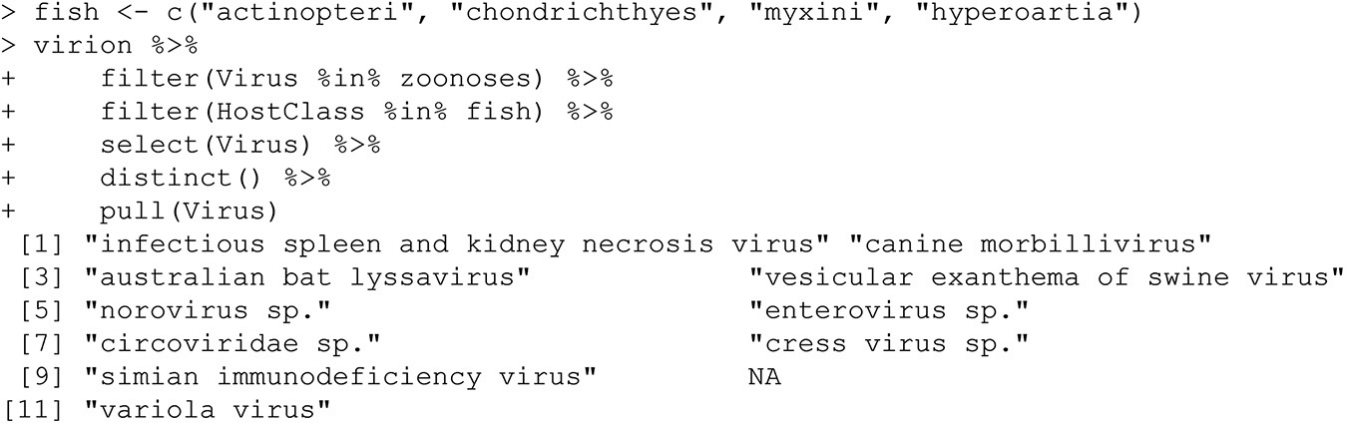


Four of these are unresolved at the species level (despite valid species-level identifiers in the NCBI taxonomy); these include classic foodborne pathogens found in sewage (enteroviruses and noroviruses) and poorly characterized single-stranded DNA viruses (circoviruses and circular rep-encoding single-stranded [CRESS] viruses). But the remaining six results are surprising, and each tells a slightly different story about how users should work with the types of data stored in VIRION.

### (i) Variola virus.

Variola virus—more commonly known as smallpox—is a now-eradicated human infectious disease. Although some animals have been experimentally infected, no evidence suggests that smallpox has any unknown zoonotic reservoirs or notable natural animal hosts to consider; why is this virus being reported from fish? We can start by tracing the record to the source:
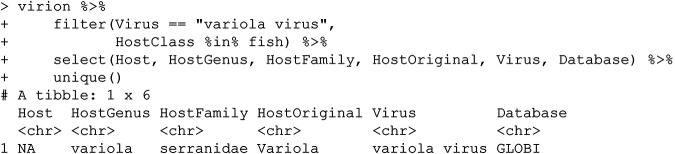


Here, GLOBI has recorded an erroneous self-interaction between this virus and itself as a “host,” likely due to an error in the processing of an automated scraping algorithm: because the record places “Variola” in the Host field, our taxonomic pipeline assigns the record to the only vertebrate host genus for *Variola*, the lyretails (Serranidae).

### (ii) Australian bat lyssavirus.

Here, we can again start by checking the source of the records that place a bat virus in a fish host:
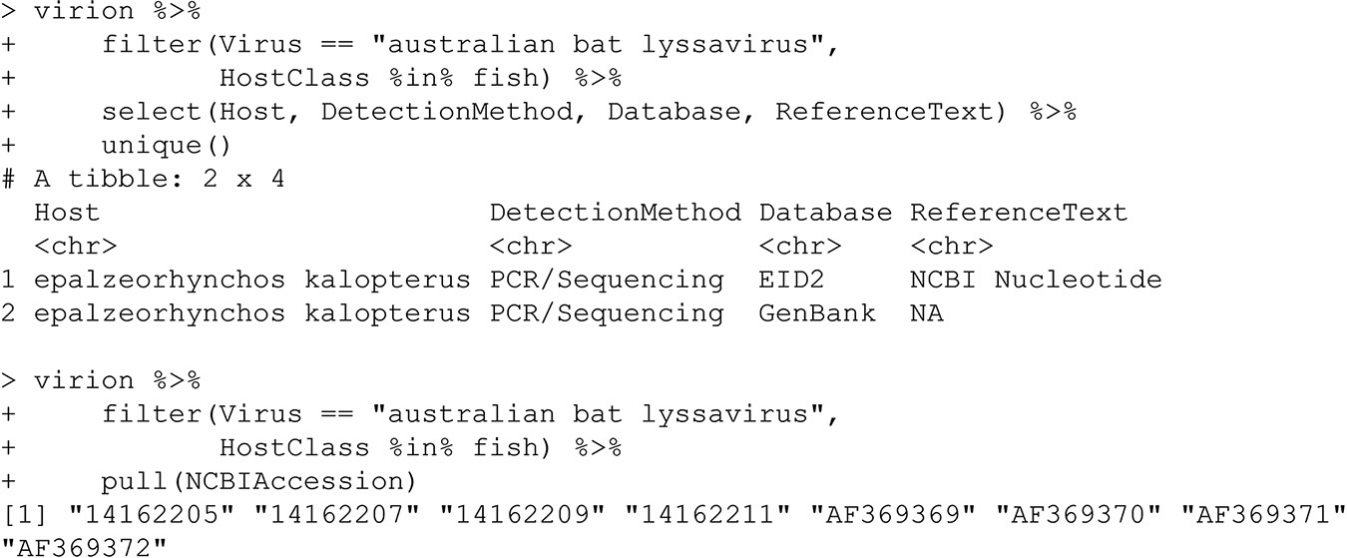


GenBank appears to be the original source for this record. There are many pitfalls that users can encounter when submitting data to sources like GenBank. What is going on with these samples?
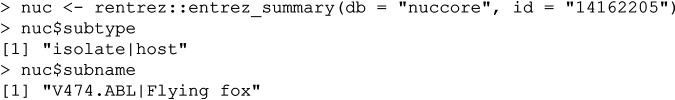


This is the result of an unfortunate ambiguity in the original submission. This host common name is a synonym in the NCBI taxonomy with the fish host Epalzeorhynchos kalopterus, which is in fact called the flying fox (TaxID: 699555); this can easily be confirmed with taxize::classification(“Flying fox", db = “ncbi”). The original data should instead refer to the flying fox bat by its scientific name (*Pteropus* sp.).

### (iii) Simian immunodeficiency virus.

This record comes from a similar problem with user-submitted ambiguous data. Here, a user has submitted the host name “Rita,” which has unfortunately matched the fish genus *Rita* (TaxID: 337744):
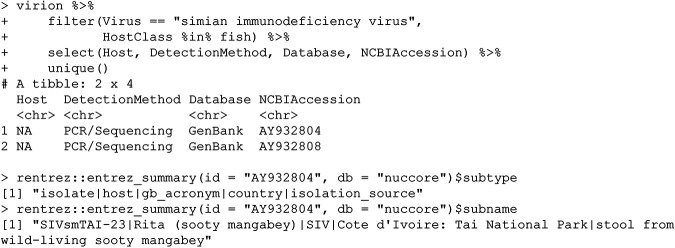


This is a bit of a strange error, given that *Rita* is not an alternate genus or outdated genus name for this species (Cercocebus atys). Who, or what, is Rita? A quick inspection of a related publication (15) reveals that Rita was the name assigned to an adult female monkey from a monitored population of sooty mangabeys in Côte d’Ivoire.

### (iv) Infectious spleen and kidney necrosis virus.

This record has a slightly different kind of problem: this is a well-studied fish virus but not one ever known to cause human disease. Why is it included on our list of zoonotic viruses? This also appears to be an erroneous GenBank accession, which—as with the previous example—has been propagated elsewhere:
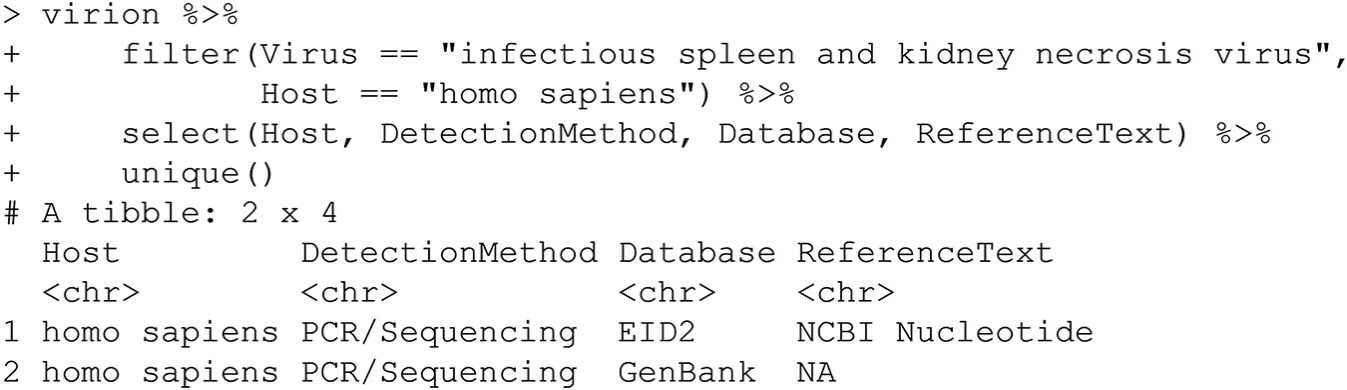


The host assigned to the relevant accessions (e.g., HQ317457.1) is Homo sapiens, for reasons that cannot be easily reconstructed; the source publication (16) records only samples from fish species. This may be the result of an automated pipeline error or something similar but is difficult to resolve and would be a case in which to directly contact the authors of these samples with further queries.

### (v) Canine morbillivirus.

These records also highlight the challenges of user-submitted GenBank data but are less identifiably the result of a user-end error.
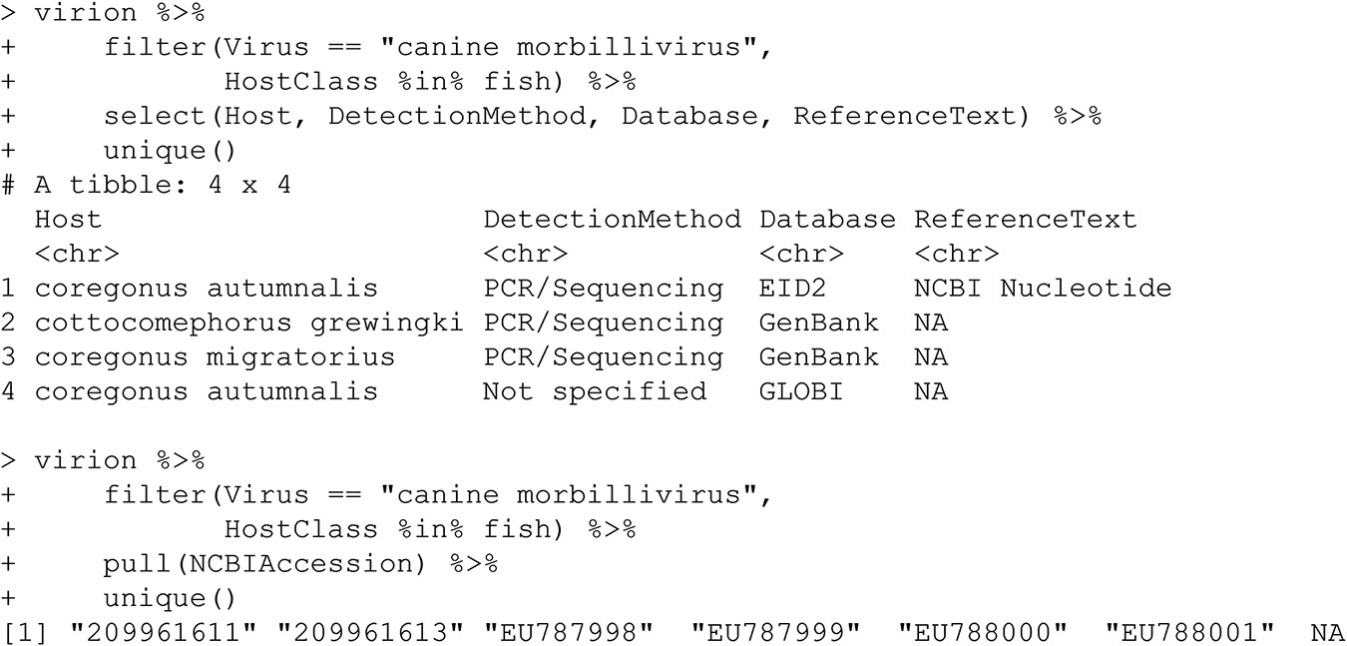


A quick check will confirm that all of these samples refer to a single study (17) on canine distemper in the Lake Baikal seal (Phoca sibirica). Though the study makes no note of samples from these species, the samples seem unlikely to be user-end errors. For example, user-submitted metadata from accession EU787998 not only record the host as “Cottocomephorus grewingki (Dybowski, 1874)” but also name the isolate as “Cottoc9-06”; these details suggest it is unlikely these entries were made in error, but without additional information from the peer-reviewed study or the authors, it is difficult to make much further inference.

### (vi) Vesicular exanthema of swine virus (VESV).

Of the six fish viruses we examined, this is perhaps the only one that seems strongly supported at face value; peer-reviewed studies have described the isolation of the virus from both the opaleye perch (18) and humans (19):

Vesicular exanthema of swine virus (VESV) is an unusual virus. The species *sensu lato* includes a number of closely related caliciviruses found in cattle, marine mammals, and even reptiles. Work from the 1990s reports that the virus can cause disease in humans (19), who appear to sometimes acquire the infection through exposure to sewage-contaminated shellfish; however, the Wikipedia entry for VESV currently states that the virus is “not transmissible to humans,” highlighting the value of VIRION as a resource to point to these older records. Given how many viruses are classified under VESV at the species rank, it may be that the lineages that infect fish and humans are quite genetically distinct and may eventually be reclassified as separate species; currently, the ICTV recognizes VESV and feline calicivirus as the two valid species in the genus (*Caliciviridae*: *Vesivirus*), as well as a few other unclassified viruses. For now, among the possible candidates for a “fish-borne zoonotic virus” in VIRION, VESV is certainly the strongest contender.

### Anticipated future improvements.

We aim to maintain and expand VIRION to keep pace with the rapidly emerging field of viral ecology. The existing data set has notable opportunities for future metadata improvement; for example, efforts to manually or programmatically revisit source publications may help flag experimental infections and better differentiate host-virus associations that are merely biologically possible from those that already occur in nature. Similarly, future iterations of the data set might incorporate new kinds of taxonomic designations, particularly if it becomes possible to efficiently implement OTU-based designations from sequence data at scale. In all of these cases, researchers can expect minor announcements about changes in workflow, scope, and format to be made via GitHub release notes, while more sizable updates could also include a blog post on the website of the Verena Consortium (viralemergence.org) or, in the case of major expansions, an additional scientific publication.

Keeping VIRION useful to researchers will also require ongoing efforts to better integrate existing sources (e.g., the continuously updated EID2 database, if the web interface is updated to allow full downloads) and new sources developed in the future. VIRION is intended to be the flagship database at the heart of a broader open data ecosystem developed by the Verena Consortium and will eventually be integrated with other existing databases (e.g., the bat betacoronavirus host database [viralemergence.org/betacov]) and other data sets yet to be developed. The current scope of VIRION could also lay the foundation to incorporate other data sources beyond vertebrate viruses; for example, we have developed the ‘insectDisease’ R package to access the Ecological Database of the World’s Insect Pathogens (20), but some of these data could be easily incorporated into a future iteration of VIRION that includes all animals. Similarly, a specialized extension of VIRION may be useful to describe vector-arbovirus relationships, which have been catalogued in a few publications (e.g., reference 21) and may benefit from tailored data architectures. There may even come a point where researchers or teams (e.g., broadly coordinated efforts to discover wildlife viruses) may want to deposit association data as they are gathered directly into VIRION, particularly if these data are used to power model-guided sampling strategies that could accelerate viral discovery (22). All of these may be future priorities for VIRION development.

## MATERIALS AND METHODS

### Data sources and tailored curation.

VIRION aggregates information from a total of five static sources (referred to as HP3, GMPD2, EID2, Shaw, and PREDICT) and two dynamic sources (Global Biotic Interactions and GenBank, hosted by the National Center for Biotechnology Information [NCBI]). The first four of these sources are separately reconciled in a new version of the CLOVER database (6), which previously covered only mammalian viruses but is here expanded to all vertebrate hosts; the rest of the data sources are integrated into the same data architecture. There is a high degree of redundancy among these different sources, both because they draw on the same limited body of scientific literature and because they may directly query or incorporate each other (e.g., many of the viral genome sequences generated by the PREDICT program have been deposited in GenBank). However, there are also significant differences in coverage between these data sources, and their reconciliation can reduce data sparsity by 30% or more (6).

### (i) The expanded CLOVER database.

In a previous study (6), we presented a semimanually curated data set called CLOVER (version 0.1.0) that reconciled four sources of information on mammal-virus interactions: the Host-Pathogen Phylogeny Project (HP3) database (23), the Global Mammal Parasite Database 2.0 (24), the static version of the ENHanCEd Infectious Disease Database (EID2) from its original open release (25), and an unnamed data set developed by Shaw et al. referred to by its lead author (26). Each of these data sets has been widely used for hypothesis testing in viral macroecology and zoonotic risk assessment. In the updated CLOVER data set (version 1.0.0) that we introduce here, the purview of this reconciliation is expanded to all vertebrate hosts available in the four databases (two of which, EID2 and Shaw, include nonmammalian hosts). As with CLOVER, three of these databases (HP3, GMPD2, and Shaw) are static sources, while EID2 is dynamically updated but cannot be downloaded in a comprehensive format, limiting our efforts to the 2015 static release (25).

The reconciliation process was the same as described in the original CLOVER publication (6). First, virus names were manually standardized across all four data sets to ensure consistency in naming convention. Then we used the R package ‘taxize’ (27) to automatically match all host and virus names to the NCBI taxonomic backbone and access higher taxonomy. Remaining host and virus names without an exact match were then manually reconciled. A percentage of 99.3% of hosts (*n* = 2,327) and 97.7% of viruses (*n* = 973) were matched to a taxonomic identifier in the NCBI database; higher taxonomy for the remaining unmatched 16 hosts and 23 viruses was manually curated by a comparison against other data sources (the IUCN Red List database and a mammal phylogenetic supertree [28]). Virus detection methods as reported for each association were standardized to a 4-tier classification system: either (i) antibodies (i.e., serological evidence), (ii) genetic sequencing (PCR or related methods), (iii) viral isolation, or (iv) not reported. Temporal metadata (year of accession or publication) were accessed for each association, either from the reported primary source publication or by querying associated PubMed and NCBI Nucleotide records using the R package ‘rentrez’ (29). The reconciled CLOVER data set contained a total of 70,466 records comprising 8,004 unique host-virus associations.

### (ii) The USAID PREDICT testing database.

The USAID Emerging Pandemic Threats (EPT) PREDICT program ran from 2009 to 2019 and remains the largest coordinated program of wildlife viral discovery to date, with a roughly $200 million U.S. dollar (USD) investment that led to the identification of 218 known and 931 novel viruses (30, 31). In 2021, data generated by this program were released on the USAID data library, and during the project’s period of performance, were also periodically released through HealthMap (healthmap.org/predict), with some slight differences between the two versions of the dataset. Many, but not all, of these sequences have been uploaded to GenBank (see below), while others are already present in literature-based databases, but a number of novel records are also given in these newly released data.

We obtained all the data available through the HealthMap public API, which includes a slightly greater coverage of data than the USAID data library release. We then downloaded the USAID version of the data library once released and added any associations not present in the other data set. All host names were already cleaned and formatted for reuse, though a few records were manually flagged for uncertain host identification; in PREDICT studies, some hosts were identified solely by relying on morphological traits observed in the field, while others had identification confirmed via genetic barcoding. Some of these are denoted by an asterisk in the HealthMap data copy, while others are given as “Genus cf. species” in both data copies; in both cases, we retained this self-reported information in the ‘HostFlagID’ field as a measure of uncertainty.

Virus names required a more detailed reconciliation workflow. First, virus names were simplified (i.e., influenza virus sequence details or similar lineage information was dropped). Subsequently, we validated virus names first by querying the NCBI taxonomy directly (using the ‘taxize’ R package), and subsequently by retrieving the metadata for GenBank accessions that are reported in the PREDICT data set (using the ‘rentrez’ R package). A number of names still remain unresolved, including those that follow the bespoke naming convention used by the PREDICT program. These names are assigned the lowest possible rank of higher taxonomy based on their naming convention; for example, PREDICT_MAstV-161 is assigned to the genus *Mamastrovirus* (*Stellavirales*: *Astroviridae*), while PREDICT_CoV-24 is assigned only to the family *Coronaviridae* (*Nidovirales*). For many of the viruses discovered by the PREDICT project, additional genus information is available from the SpillOver Risk Ranking Tool database (spillover.global [30]). We downloaded these data and matched them to the names in the PREDICT data releases where possible. Future efforts may be able to improve taxonomic resolution for the remaining viruses based on phylogenetic analysis using currently unreleased sequence data; these will be incorporated through GenBank accessions, rather than updates to the PREDICT data sets, which are currently presumed to be static releases.

### (iii) Global Biotic Interactions (GLOBI).

GLOBI is a dynamic, content-aggregating, open-access database of ecological interactions (32). The data in GLOBI are automatically pulled and manually curated from published scientific literature and other databases, including the bat virus database DBatVir (www.mgc.ac.cn/DBatVir), the rodent virus database DRodVir (www.mgc.ac.cn/DRodVir), and Virus-Host DB (www.genome.jp/virushostdb). As such, GLOBI collates a number of data sources with value for describing the global virome. However, in GLOBI, each species’ taxonomic identity is linked to the initial taxonomic database to which it was reconciled, representing a number of different backbones (e.g., NCBI, GBIF, and Encyclopedia of Life), each of which coexists in these data sets. As such, these data may include duplications, outdated synonyms, or other inconsistencies: for example, “Virus” and “Viruses” are both used as high-level ranks.

We use the R package ‘rglobi’ (33) to automatically download all associations between viruses (queried as both “Virus” and “Viruses”) and vertebrates (queried as “Vertebrata”) in the data set. Virus names are simplified (i.e., influenza A/B/C/D sequence details or similar lineage information is dropped), and both host and virus names are validated against the NCBI taxonomic backbone. In an effort to standardize data quality, we retain only hosts that can be matched (and confirmed as vertebrates) against the NCBI backbone.

### (iv) NCBI GenBank.

GenBank is the gold-standard, open-access, community-generated data set of nucleotide and protein sequence data for life on Earth, with over two billion sequences submitted as of February 2021. For viruses, there are nearly five million distinct accessions of nucleotide sequences, which can be directly downloaded from the NCBI Virus interface (https://www.ncbi.nlm.nih.gov/labs/virus) or from an FTP server (https://ftp.ncbi.nlm.nih.gov/genomes/Viruses/AllNuclMetadata/). Though the majority of viral samples are from Homo sapiens, and many others are from bacteriophages, GenBank is still an incomparable source of broad information on vertebrate-virus associations, with genetic sequence representing strong evidence in support of these associations.

For the VIRION workflow, we download the entirety of NCBI Virus’s nucleotide records from the FTP server. To ensure data quality, we limited the records incorporated into VIRION to those that had a confirmed match to a vertebrate host. (While virus samples in GenBank are almost all resolved to the NCBI taxonomic backbone, host information is contributed by users directly as sample metadata and is less standardized.)

### Data integration and taxonomic reconciliation.

We developed a reproducible, open pipeline to integrate these seven data sources in the statistical software ‘R’. Throughout, we used a standardized set of tools and procedures, relying heavily on a set of tools for biological data science developed by the ROpenSci and EcoJulia programs.

The primary component of the data is a list of hosts and viruses known to associate with each other. Where possible, these are resolved down to the species level for both (in the fields “Host” and “Virus”); otherwise, information is given down to the lowest possible valid taxonomic rank (e.g., *Betacoronavirus* in “VirusGenus” or Chiroptera in “HostClass”), and original entries are retained (“HostOriginal,” “VirusOriginal”). All of these fields are stored in all-lowercase letters to avoid inconsistencies in capitalization. Information is also recorded on whether species names are considered valid in the NCBI and International Committee on the Taxonomy of Viruses (ICTV) taxonomies and whether host identification was originally reported as uncertain.

Unlike other data sources that reproduce taxonomic information verbatim or use different taxonomic backbones on a per-data-point basis (e.g., GLOBI), VIRION is fully reconciled against the taxonomic hierarchy curated by the NCBI for both animals and viruses. To do so, we use the R package ‘taxize’ (27) to access the NCBI API and—for larger tasks—an R wrapper (available in the VIRION repository) for the Julia package ‘NCBITaxonomyjl’ (34), which executes local matching that can be multiple orders of magnitude faster. For animal hosts, the classification of major lineages generally follows the broad consensus of names selected under the appropriate code of nomenclature in recent systematic studies, while taking a conservative approach to areas that remain contentious or unresolved. For viruses, the taxonomic hierarchy is dynamically updated (and these updates propagated) to be fully harmonized with the contemporary decisions of the ICTV; although the parsing and mapping of new names to the taxonomic backbone are done automatically, the names are propagated only after review by the NCBI Taxonomy group. In addition, users can raise issues of taxonomic mismatch between NCBI and ICTV directly with the NCBI Taxonomy group (suggest@ncbi.nlm.nih.gov), which allows these issues to be solved based on community feedback when supported by an authority; this ensures that issues identified by the community do not overrule ICTV or ICZN.

In addition to taxonomic information, a minimum standard of metadata is retained from all sources (Table 1) and integrated in a data harmonization pipeline modeled off the CLOVER pipeline. This includes information on the source of the data; the date of sample collection, data release, or related scientific publication; detection methods; and other citation-related or accession-related information.

**TABLE 1:** Data field descriptors for the VIRION database

Data field (column)	Data type	Descriptor
Host, HostGenus, HostFamily, HostOrder, HostClass	Character string	Host taxonomy, including higher taxonomy (all lowercase).
Virus, VirusGenus, VirusFamily, VirusOrder, VirusClass	Character string	Virus taxonomy, including higher taxonomy (all lowercase).
ICTVRatified	Boolean	Is the virus species given in the field “Virus” considered a valid species name in the latest ICTV taxonomy?
HostNCBIResolved, VirusNCBIResolved	Boolean	Is the lowest nonmissing taxonomic value (usually species level but sometimes higher) matched to the NCBI taxonomy?
HostTaxID, VirusTaxID	Numeric character string	The “TaxID” unique identifier to the lowest possible taxonomic match in the NCBI database; in some cases this may be below the lowest taxonomic resolution (e.g., some virus species may have an NCBI identifier below the species level).
HostOriginal, VirusOriginal	Character string	Original entry for host and virus taxonomy as provided in source database (verbatim and not necessarily lowercase).
HostFlagID	Boolean	Values are given as TRUE if source metadata reports any uncertainty in host identification (e.g., “cf.” in a species name or the flags in the PREDICT data).
DetectionMethod, DetectionOriginal	Character string	DetectionMethod harmonizes four categories (in descending order of strength of evidence: “Isolation/Observation,” “PCR/Sequencing,” “Antibodies,” and “Not specified”) from the raw information provided in DetectionOriginal. Harmonized values are given to the highest evidence level possible based on a source record (e.g., the plaintext value “Isolation and antibodies” is harmonized to “Isolation/Observation”). In some cases where detection method is not available via metadata, source information is used as DetectionOriginal (e.g., “NCBI Nucleotide”).
Database, DatabaseVersion	Character string	Data provenance traces back to seven source data sets (EID2, Shaw, HP3, GMPD2, PREDICT, GenBank, and GLOBI), with linked information about either the citation of the relevant copy (e.g., “Shaw et al. 2020 Mol Ecol”) or version information for dynamic sources (e.g., “Aug2021FlatFile” for GenBank).
ReferenceText, PMID, PublicationYear	Character string, character string, numeric	Bibliographic information is sourced from the CLOVER database for records derived from Shaw, GMPD2, HP3, or EID2. ReferenceText provides a text description of literature sources (where provided in source data sets); PMID provides PubMed identifiers for literature sources (where provided); PublicationYear provides the year the literature source was published, accessed either from the original database’s reference description or from scraping the PubMed database.
NCBIAccession	Character string	NCBI accession information is given for records that originate directly from GenBank or have other linked metadata in other sources, including both the CLOVER data and the PREDICT data. These can be readily used in combination with tools like ‘rentrez’ to query source information on viral samples.
CollectionDay, CollectionMonth, CollectionYear	Numeric	Reports the date of actual sample collection (not the release of data or a published paper) as provided for samples from PREDICT or GenBank
ReleaseDay, ReleaseMonth, ReleaseYear	Numeric	Reports the year a given association was “released” in public information (EID2 and PREDICT) or a publicly deposited sample on GenBank. For PREDICT, all values are given as 2021, given the release of a static file at that time even though some findings may have been published or deposited in GenBank earlier. (This redundancy should be captured in overlap with GenBank and EID2.)

### Data and code availability.

The VIRION database, and the code used to generate it, is available from the Verena Consortium’s GitHub account at github.com/viralemergence/virion. Additional code used to produce the CLOVER data set is available at github.com/viralemergence/clover.
